# Cardiac Surgery-associated Acute Kidney Injury in Patients with Preserved Baseline Renal Function

**DOI:** 10.21470/1678-9741-2022-0108

**Published:** 2022

**Authors:** Patrícia Silva Marco, Marcelo Arruda Nakazone, Lilia Nigro Maia, Maurício Nassau Machado

**Affiliations:** 1 Department of Cardiology and Cardiovascular Surgery, Faculdade de Medicina de São José do Rio Preto, São José do Rio Preto, São Paulo, Brazil; 2 Postgraduate Division, Faculdade de Medicina de São José do Rio Preto, São José do Rio Preto, São Paulo, Brazil; 3 Hospital de Base, Fundação Faculdade Regional de Medicina de São José do Rio Preto, São José do Rio Preto, São Paulo, Brazil

**Keywords:** Acute Kidney Injury, Cardiac Surgical Procedures, Estimated Glomerular Filtration Rate, Renal Replacement Therapy, Mortality

## Abstract

**Introduction:**

Cardiac surgery-associated acute kidney injury (CSA-AKI) is a powerful predictor of perioperative outcomes. We evaluated the burden of CSA-AKI in patients with preserved baseline renal function.

**Methods:**

The data of 2,162 adult patients who underwent cardiac surgery from January 2005 to December 2020 were analyzed. Logistic regression models were used to determine predictors of CSA-AKI and their associations with hospital mortality up to 30 days.

**Results:**

The prevalence of acute kidney injury was 43.0%, and 2.0% of patients required renal replacement therapy. Hospital mortality rate was 5.6% (non-acute kidney injury = 2.0% vs. CSA-AKI = 10.4%, P<0.001), and any degree of CSA-AKI was associated with a significant increase in death rates (stage 1 = 4.3%, stage 2 = 23.9%, stage 3 = 59.7%). Multivariable logistic regression analysis identified age, obesity, left ventricular dysfunction, previous cardiac surgery, and cardiopulmonary bypass duration as predictors of CSA-AKI. Moreover, CSA-AKI was confirmed as independent predictor of hospital mortality for stage 1 (odds ratio, 2.02; 95% confidence interval, 1.16 to 3.51; P=0.013), stage 2 (odds ratio, 9.18; 95% confidence interval, 4.54 to 18.58; P<0.001), and stage 3 (odds ratio, 37.72; 95% confidence interval, 18.87 to 75.40; P<0.001) patients.

**Conclusion:**

Age, obesity, left ventricular dysfunction, previous cardiac surgery, and cardiopulmonary bypass duration are independent predictors of CSA-AKI in patients with preserved baseline renal function. The development of CSA-AKI is significantly associated with worse outcomes, and there is a dose-response relationship between acute kidney injury stages and hospital mortality.

**Table:** 

Abbreviations, Acronyms & Symbols				
AKD	= Acute kidney disease		eGFR	= Estimated glomerular filtration rate
AKI	= Acute kidney injury		HVS	*=* Heart valve surgery
BMI	= Body mass index		ICU	= Intensive care unit
CABG	= Coronary artery bypass grafting		KDIGO	= Kidney Disease Improving Global Outcomes
CI	= Confidence interval		LoS	= Length of stay
CKD	= Chronic kidney disease		LVEF	= Left ventricular ejection fraction
CKD-EPI	= Chronic Kidney Disease Epidemiology Collaboration		OR	= Odds ratio
COPD	= Chronic obstructive pulmonary disease		RRT	= Renal replacement therapy
CPB	= Cardiopulmonary bypass		SCr	= Serum creatinine
CSA-AKI	= Cardiac surgery-associated acute kidney injury			

## INTRODUCTION

Preoperative renal dysfunction is common in the cardiac surgery population, and operative mortality rises inversely with declining renal function. Preoperative estimated glomerular filtration rate (eGFR) is one of the most powerful predictors of operative mortality and morbidity^[1]^, as well as cardiac surgery-associated acute kidney injury (CSA-AKI)^[2]^.

The severity of CSA AKI ranges from asymptomatic to requiring renal replacement therapy (RRT)^[3]^, and there is a significant variation in the reported incidence of CSA-AKI, ranging from 9.25% to 49.25%^[4,5]^, as well as in the mortality rate in no acute kidney injury (AKI) (0.4% to 2.8%)^[4,6]^ and in AKI patients (4.6% to 24.2%)^[7,8]^, which can be explained by the different AKI definitions used in the studies. Among these patients, up to 2% to 6% require RRT^[9]^, which is linked to the risk of short- and long-term adverse events and increases the costs of postoperative treatment^[10,11]^. The proportion of patients with complete renal function recovery at discharge declines gradually with increasing AKI severity^[6]^. CSA-AKI can also predict the development of chronic kidney disease (CKD) in the future^[11]^.

Since even small changes in serum creatinine (SCr) are associated with increased early mortality^[12,13]^ and because AKI staging remains an independent predictor of death, with robust results in patients with preserved baseline renal function^[11,14]^, the aim of this study was to assess hospital mortality up to 30 days and clinical outcomes associated with the development of CSA-AKI in patients with preserved baseline renal function (eGFR by the Chronic Kidney Disease Epidemiology Collaboration [CKD-EPI] equation^[15]^ ≥ 60 mL/min/1.73 m^2^) using the Kidney Disease Improving Global Outcomes (KDIGO) classification^[16]^.

## METHODS

### Patient Selection

This is a single-center study retrospectively evaluating patients who underwent cardiac surgery in a Brazilian Medical School facility. The demographics, type of surgery, laboratory data, and preoperative, perioperative, and postoperative information were retrieved from a prospectively collected database of 3,799 adult patients who underwent cardiac surgery from January 2005 to December 2020. All patients were operated on by the same surgical team using cardiopulmonary bypass (CPB) with a Medtronic® centrifugal pump and membrane oxygenator, heat exchanger, and cardiotomy reservoir by Braile Biomédica®. Cardioplegia was usually performed with a Custodiol, Del Nido, or Buckberg solution (4:1 isothermal blood), varying its use and application (antegrade or retrograde) according to the type of surgery and proposed treatment strategy.

**Fig. 1:**
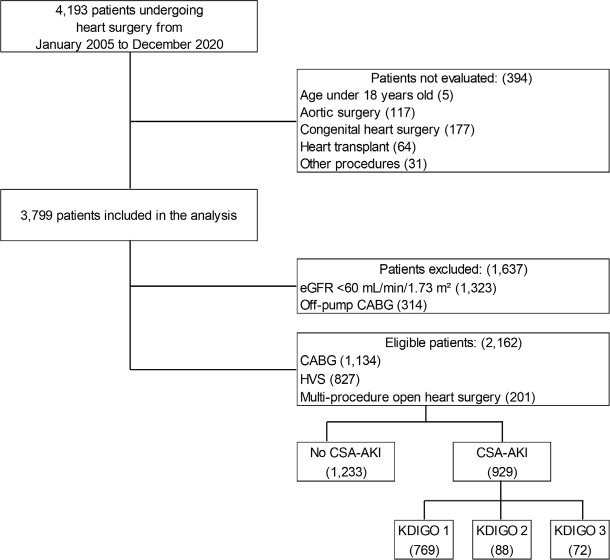
Study flow chart showing patients who underwent cardiac surgical procedures and who were divided into two groups according to the development of cardiac surgery-associated acute kidney injury (CSA-AKI) based on the Kidney Disease Improving Global Outcomes (KDIGO) classification. CABG=coronary artery bypass grafting; eGFR=estimated glomerular filtration rate; HVS=heart valve surgery.

After exclusions (1,323 patients with baseline eGFR < 60 mL/min/1.73 m^2^ and 314 patients who underwent off-pump coronary artery bypass grafting [CABG]), a total of 2,162 patients were suitable for analysis: 1,134 (52.0%) patients who underwent CABG, 827 (38.0%) patients who underwent heart valve surgery (HVS), and 201 (9.0%) patients who underwent multi-procedure open heart surgery (Figure 1). The patients were divided into two groups according to the development of CSA-AKI based on the KDIGO classification^[16]^. Subsequently, a subgroup was analyzed based on the three stages of AKI severity.

This study was conducted in accordance with the 1975 Declaration of Helsinki, revised in 2013 by the World Medical Association, and the Brazilian National Health Council Resolution 466/2012 and approved by the Local Human Research Ethics Committee of Faculdade de Medicina de São José do Rio Preto (CAAE: 44844321.2.0000.5415). The need for individual informed consent was waived, as this study was a retrospective analysis of prospectively collected data for routine care, and there was no breach of privacy or anonymity. The methodology of this investigation is consistent with the STROBE checklist for observational studies.

### Serum Creatinine Measurement

The Jaffe colorimetric method (ADVIA 1650, Bayer, Germany) was used to measure SCr concentration. The reference values are 0.6 to 1.3 mg/dL for adult men and 0.6 to 1.0 mg/dL for adult women. The CKD-EPI equation^[15]^ was then applied to estimate the glomerular filtration rate (mL/min/1.73 m^2^) using the baseline SCr, and the KDIGO classification^[16]^ was used for diagnosis and staging of CSA-AKI.

### Diagnosis and Staging of AKI (KDIGO classification)^[16]^

In our series, AKI was defined as any of the following:

- Increase in SCr by ≥ 0.3 mg/dL (≥ 26.5 µmol/L) within 48 hours or
- Increase in SCr to ≥ 1.5 times baseline, which is known or presumed to have occurred within the prior seven days

AKI was also staged for severity according to the following criteria based on SCr:

- Stage 1: 1.5 to 1.9 times baseline or ≥ 0.3 mg/dL (≥ 26.5 µmol/L) increase
- Stage 2: 2.0 to 2.9 times baseline
- Stage 3: 3.0 times baseline or increase in SCr to ≥ 4.0 mg/dL (≥ 353.6 µmol/L) or initiation of RRT

### Outcomes

The primary objective of this study was to determine the association between CSA-AKI and hospital mortality up to 30 days, and the secondary objective was to identify predictors of CSA-AKI. Other clinical outcomes evaluated were: the need for RRT, reoperation due to bleeding/cardiac tamponade, acute atrial fibrillation, reintubation up to seven days after surgery, prolonged mechanical ventilation (> 24 hours), operating room extubation, type I neurological injury (new episode of motor deficit, coma, seizure or encephalic lesion documented by cranial computed tomography or magnetic resonance imaging), intensive care unit (ICU) readmission rate, ICU length of stay (up to 30 days), and prolonged ICU stay (> 14 days).

### Statistical Analysis

Categorical data are presented as absolute numbers and percentages, and continuous variables as median and interquartile ranges (25^th^ and 75^th^ percentiles). Continuous variables were compared using the nonparametric Mann-Whitney or Kruskal-Wallis tests. Chi-square or Fisher’s exact tests were used to compare categorical variables.

Univariate and multivariable logistic regression models (enter elimination method) were used to identify predictors of CSA-AKI and determine the association between CSA-AKI and hospital mortality up to 30 days. Both models were adjusted for age (years), sex (reference: male), obesity (body mass index [BMI] ≥ 30 kg/m^2^), chronic obstructive pulmonary disease (COPD), hypertension, diabetes mellitus, baseline eGFR (mL/min/1.73 m^2^), left ventricular ejection fraction (LVEF) ≤ 30%, previous cardiac surgery, urgent/emergency surgery, multi-procedure open heart surgery, and CPB duration (min). Additionally, we added the staging of renal function (KDIGO 1 to 3) to the hospital mortality model (reference: no AKI) to identify its independent predictors.

The purposeful selection process began with a univariate analysis of each of the variables abovementioned. Any variable having a univariate test with a *P*-value < 0.10 was selected as a candidate for multivariable analysis. All variables included in the multivariable regression models were tested for multicollinearity using the variance inflation factor. The adjusted odds ratio (OR) and 95% confidence intervals (CI) were calculated for the predictors.

The data were analyzed using the IBM Corp. Released 2019, IBM SPSS Statistics for Windows, version 26.0, Armonk, NY: IBM Corp. *P*-values < 0.05 were considered statistically significant (two-tailed).

## RESULTS

The demographic data, risk factors, and renal and left ventricular function for each patient are described in Table 1. Surgery status, risk score, clinical outcomes, discharge, and hospital mortality up to 30 days are presented in Table 2. The CSA-AKI patients were older and had a higher proportion of obesity, hypertension, and diabetes mellitus. There was no difference in baseline SCr, but eGFR was slightly lower in patients who developed CSA-AKI. This group of patients also had a greater proportion of severe left ventricular dysfunction (Table 1). Patients who developed CSA-AKI had worse results in every outcome evaluated (Table 2).

**Table 1:** Baseline characteristics and renal and left ventricular function of the 2,162 patients according to the development of CSA-AKI based on KDIGO criteria.

	All patients n = 2,162	No CSA-AKI n = 1,233	CSA-AKI n = 929	*P*-value
**Demographics and hospitalization**				
Age (years)	57 (48 - 64)	55 (46 - 62)	59 (51 - 67)	< 0.001
18 to < 40	248 (11.5)	183 (14.9)	65 (7.0)	< 0.001
40 to < 65	1396 (64.7)	820 (66.7)	576 (62.1)	0.029
65 to < 75	443 (20.5)	206 (16.7)	237 (25.6)	< 0.001
≥ 75	70 (3.2)	21 (1.7)	49 (5.3)	< 0.001
Male sex	1427 (66.0)	803 (65.1)	624 (67.2)	0.321
**Risk factors**				
Weight (kg)	72 (63 - 82)	72 (63 - 82)	73 (62 - 83)	0.771
Height (m)	1.66 (1.60 - 1.72)	1.67 (1.60 - 1.72)	1.65 (1.60 - 1.72)	0.126
Body mass index (kg/m^2^)	26 (24 - 29)	26 (24 - 29)	26 (23 - 30)	0.336
Normal weight (18.5 to < 25.0)	794 (36.7)	455 (36.9)	339 (36.5)	0.884
Low weight (< 18.5)	54 (2.5)	31 (2.5)	23 (2.5)	0.955
Overweight (25.0 to < 30.0)	843 (39.0)	504 (40.9)	339 (36.5)	0.039
Obesity (≥ 30.0)	471 (21.8)	243 (19.7)	228 (24.5)	0.007
Body surface area^*^ (m^2^)	1.80 (1.66 - 1.95)	1.80 (1.66 - 1.95)	1.80 (1.66 - 1.94)	0.750
Chronic obstructive pulmonary disease	68 (3.1)	36 (2.9)	32 (3.4)	0.489
Hypertension	1387 (64.2)	761 (61.7)	626 (67.4)	0.007
Diabetes mellitus	473 (21.9)	250 (20.3)	223 (24.0)	0.038
**Renal and left ventricular function**				
Baseline serum creatinine (mg/dL)	1.0 (0.8 - 1.1)	1.0 (0.8 - 1.1)	1.0 (0.8 - 1.1)	0.303
Baseline eGFR (mL/min/1.73 m^2^)	78 (68 - 91)	79 (69 - 92)	78 (67 - 91)	0.041
**Left ventricular ejection fraction (%)**				
> 50	1758 (81.3)	1030 (83.5)	728 (78.4)	0.002
31 to 50	254 (11.7)	136 (11.0)	118 (12.7)	0.232
≤ 30	150 (6.9)	67 (5.4)	83 (8.9)	0.002

Values are presented as n (%) for categorical data or as median with first and third quartiles for scalar values.

^*^DuBois & DuBois method.

CSA-AKI=cardiac surgery-associated acute kidney injury; eGFR=estimated glomerular filtration rate; KDIGO=Kidney Disease Improving Global Outcomes

**Table 2:** Surgery status, risk score, and clinical outcomes of the 2,162 patients according to the development of CSA-AKI based on KDIGO criteria.

	All patients n = 2,162	No CSA-AKI n = 1,233	CSA-AKI n = 929	*P*-value
**Surgery status, risk score, and clinical outcomes**				
Previous cardiac surgery	216 (10.0)	92 (7.5)	124 (13.3)	< 0.001
Urgent/emergency surgery	629 (29.1)	340 (27.6)	289 (31.1)	0.073
InsCor^*^	2 (0 - 5)	2 (0 - 4)	3 (2 - 5)	< 0.001
Low risk	1290 (63.4)	798 (68.3)	492 (56.7)	< 0.001
Intermediate risk	576 (28.3)	305 (26.1)	271 (31.3)	0.011
High risk	169 (8.3)	65 (5.6)	104 (12.0)	< 0.001
Cardiopulmonary bypass duration (min)	92 (76 - 111)	90 (75 - 105)	97 (79 - 120)	< 0.001
< 90	916 (43.8)	578 (48.0)	338 (38.1)	< 0.001
90 to < 120	786 (37.6)	460 (38.2)	326 (36.8)	0.498
≥ 120	389 (18.6)	166 (13.8)	223 (25.1)	< 0.001
**Type of surgery**				
Coronary artery bypass grafting	1230 (56.9)	712 (57.7)	518 (55.8)	0.356
Heart valve surgery	1016 (47.0)	559 (45.3)	457 (49.2)	0.075
Multi-procedure open heart surgery	201 (9.3)	100 (8.1)	101 (10.9)	0.029
**Postoperative outcomes**				
Renal replacement therapy up to 7 days	40 (1.9)	0 (0.0)	40 (4.3)	< 0.001
Reoperation for bleeding/tamponade	76 (3.5)	24 (1.9)	52 (5.6)	< 0.001
Acute atrial fibrillation	232 (10.7)	96 (7.8)	136 (14.6)	< 0.001
Tracheal reintubation up to 7 days	136 (6.3)	28 (2.3)	108 (11.6)	< 0.001
Prolonged pulmonary ventilation (> 24 hours)	227 (10.5)	42 (3.4)	185 (19.9)	< 0.001
Operating room extubation	746 (34.5)	490 (39.7)	256 (27.6)	< 0.001
Type 1 neurological injury*†*	95 (4.4)	33 (2.7)	62 (6.7)	< 0.001
**Discharge and mortality**				
ICU readmission	122 (5.6)	52 (4.2)	70 (7.5)	0.001
ICU LoS up to 30 days	3 (2 - 5)	2 (2 - 4)	4 (2 - 7)	< 0.001
Long LoS (> 14 days)	122 (5.6)	26 (2.1)	96 (10.3)	< 0.001
Hospital mortality up to 30 days	122 (5.6)	25 (2.0)	97 (10.4)	< 0.001

Values are presented as n (%) for categorical data or as medians with first and third quartiles for scalar values.

^*^InsCor is a Brazilian risk assessment model for patients undergoing coronary artery bypass grafting (CABG) and/or heart valve surgery (HVS) that uses 10 variables: age > 70 years; female sex; combined surgery (CABG and HVS); myocardial infarction < 90 days; reoperation; aortic valve replacement; tricuspid valve repair/replacement; serum creatinine > 2 mg/dL; left ventricular ejection fraction < 30%; and events (at least one of the following situations prior to surgery: intra-aortic balloon pump, cardiogenic shock, ventricular tachycardia or fibrillation, orotracheal intubation, acute renal failure, inotropic agents use, and cardiopulmonary resuscitation). The InsCor score ranges from 0 to 30 points and defines three categories of mortality risk after cardiac surgery: low risk (0 to 3 points), intermediate risk (4 to 7 points), and high risk (≥ 8 points).

*†*Type 1 neurological injury: new episode of motor deficit, coma, seizure, or encephalic lesion documented by cranial computed tomography or magnetic resonance imaging.

CSA-AKI=cardiac surgery-associated acute kidney injury; ICU=intensive care unit; KDIGO=Kidney Disease Improving Global Outcomes; LoS=length of stay

The prevalence of AKI was 43% distributed in 36% of patients with stage 1 AKI, 4% of patients with stage 2 AKI, and 3% of patients with stage 3 AKI. A total of 40 (2%) patients required RRT, representing 56% of stage 3 patients.

### All-Cause Mortality up to 30 Days

One hundred and twenty-two patients died within the first 30 days after surgery (5.6%). Cause of death data was available for 90% of patients. Cardiovascular complication was the primary cause for 48 patients (44%), while 56% of deaths were attributed to noncardiac issues.

Patients with no AKI had a 2.0% mortality rate, while patients with CSA-AKI had a 10.4% mortality rate. Any degree of CSA-AKI was associated with a significant increase in mortality up to 30 days: 4.3% for stage 1, 23.9% for stage 2, and 59.7% for stage 3. CSA-AKI stage 3 patients who did not require RRT had a mortality rate of 59.4%, while the mortality of patients needing RRT was 60% (*P*>0.999).

Non-survivors were older (62 *vs*. 56 years old; *P*<0.001), but there were no differences between sex, hypertension, and diabetes mellitus. There was a greater proportion of non-survivors with low weight (BMI < 18.5 kg/m^2^; 6.6% *vs.* 2.3%, *P*=0.009) and COPD (7.4% *vs.* 2.9%, *P*=0.006). Non-survivors had a greater proportion of previous cardiac surgery (20.5% *vs.* 9.4%, *P*<0.001), were more frequently operated on an urgent/emergency condition (52.5% *vs.* 27.7%, *P*<0.001), and had a longer CPB duration (115 *vs.* 91 minutes, *P*<0.001). All rates of postoperative outcomes were worse in non-survivors (*P*<0.001 for all), except the rate of ICU readmission (9.0% *vs.* 5.4%, *P*=0.096) (data not shown).

In the subgroup analysis, the overall mortality of CABG, HVS, and multi-procedure open heart surgery patients was 4.4%, 5.4%, and 13.4%, respectively (*P*<0.001). Patients with no AKI had mortality rates of 1.8%, 1.7%, and 5.0%, respectively, while patients with CSA-AKI (stage 1) had 4.0%, 4.6%, and 4.3%; stage 2 presented 18.6%, 20.0%, and 46.7%; and stage 3 showed 58.3%, 54.8%, and 70.6% rates for hospital mortality up to 30 days, respectively (*P*>0.05 for all) (data not shown).

### Multivariable Logistic Regression Models

The multivariable logistic regression analysis showed that age, obesity, LVEF ≤ 30%, previous cardiac surgery, and CPB duration were independent predictors of CSA-AKI (Table 3).

**Table 3:** Multivariable analysis using logistic regression models - odds ratio (OR) and 95% confidence intervals (CI) for predictors of CSA-AKI based on KDIGO classification.

	Univariate analysis			Multivariable analysis		
	**OR**	**95% CI**	***P*-value**	**OR**	**95% CI**	***P*-value**
Age (years)	1.03	1.02 - 1.04	< 0.001	1.04	1.03 - 1.04	< 0.001
Male sex	1.10	0.92 - 1.31	0.321			
Obesity (BMI ≥ 30.0 kg/m^2^)	1.33	1.08 - 1.63	0.007	1.44	1.16 - 1.79	0.001
Chronic obstructive pulmonary disease	1.19	0.73 - 1.93	0.489			
Hypertension	1.28	1.07 - 1.53	0.007			
Diabetes mellitus	1.24	1.01 - 1.52	0.038			
Baseline eGFR (each mL/min/1.73 m^2^)	1.00	0.99 - 1.00	0.166			
LVEF ≤ 30%	1.71	1.22 - 2.38	0.002	1.49	1.05 - 2.12	0.026
Previous cardiac surgery	1.91	1.44 - 2.54	< 0.001	2.16	1.58 - 2.96	< 0.001
Urgent/emergency surgery	1.19	0.98 - 1.43	0.073			
Multi-procedure open heart surgery	1.38	1.03 - 1.85	0.029			
Cardiopulmonary bypass duration (min)	1.011	1.008 - 1.014	< 0.001	1.011	1.007 - 1.014	< 0.001

BMI=body mass index; CSA-AKI=cardiac surgery-associated acute kidney injury; eGFR=estimated glomerular filtration rate; KDIGO=Kidney Disease Improving Global Outcomes; LVEF=left ventricular ejection fraction

The multivariable logistic regression analysis also showed that age, COPD, urgent/emergency surgery, CPB duration, and CSA-AKI were independent predictors for hospital mortality up to 30 days (Table 4). In this case, CSA-AKI was confirmed as predictor of death for stage 1 (OR , 2.02; 95% CI, 1.16 to 3.51; *P*=0.013), stage 2 (OR , 9.18; 95% CI, 4.54 to 18.58; *P*<0.001), and stage 3 (OR , 37.72; 95% CI, 18.87 to 75.40; *P*<0.001) patients.

**Table 4:** Multivariable analysis using logistic regression models - odds ratio (OR) and 95% confidence intervals (CI) for predictors of hospital mortality up to 30 days after cardiac surgery.

	Univariate analysis			Multivariable analysis		
	**OR**	**95% CI**	***P*-value**	**OR**	**95% CI**	***P*-value**
Age (years)	1.03	1.02 - 1.05	< 0.001	1.02	1.00 - 1.04	0.032
Male sex	0.76	0.52 - 1.10	0.140			
Obesity (BMI ≥ 30.0 kg/m^2^)	1.07	0.70 - 1.66	0.748			
Chronic obstructive pulmonary disease	2.67	1.29 - 5.53	0.008	3.27	1.43 - 7.52	0.005
Hypertension	1.07	0.73 - 1.57	0.736			
Diabetes mellitus	1.18	0.77 - 1.80	0.456			
Baseline eGFR (each mL/min/1.73 m^2^)	0.99	0.98 - 1.00	0.173			
LVEF ≤ 30%	1.95	1.10 - 3.45	0.023			
Previous cardiac surgery	2.50	1.57 - 3.97	< 0.001			
Urgent/emergency surgery	2.88	1.99 - 4.16	< 0.001	1.98	1.27 - 3.10	0.003
Multi-procedure open heart surgery	3.05	1.93 - 4.80	< 0.001			
Cardiopulmonary bypass duration (min)	1.02	1.02 - 1.03	< 0.001	1.01	1.01 - 1.02	<0.001
**CSA-AKI**						
No AKI (reference)	1.00					
KDIGO 1	2.17	1.28 - 3.67	0.004	2.02	1.16 - 3.51	0.013
KDIGO 2	15.15	8.07 - 28.44	< 0.001	9.18	4.54 - 18.58	< 0.001
KDIGO 3	71.65	38.72 - 132.57	< 0.001	37.72	18.87 - 75.40	< 0.001

AKI=acute kidney injury; BMI=body mass index; CSA-AKI=cardiac surgery-associated acute kidney injury; eGFR=estimated glomerular filtration rate; KDIGO=Kidney Disease Improving Global Outcomes; LVEF=left ventricular ejection fraction

## DISCUSSION

In this single-center observational study involving 2,162 patients who underwent cardiac surgery with preserved baseline renal function, we found a 43% incidence of CSA-AKI with a hospital mortality rate in patients with and without CSA-AKI of 10.4% and 2.0%, respectively. We demonstrate that the development of CSA-AKI based on KDIGO classification is an independent predictor of hospital mortality up to 30 days as well as the AKI severity (stages 1 to 3). AKI patients had worse outcomes after surgery with increased rates of clinical complications, as described by other authors^[13,17]^. OR for mortality in patients with stage 1 AKI was 2.02 (95% CI, 1.16 to 3.51), confirming that small changes in SCr in postoperative cardiac surgery patients are independent predictors of mortality. We believe that even in patients with preserved baseline renal function undergoing cardiac surgery, the incidence of AKI is high and has a huge impact in postoperative outcomes as well as on hospital mortality rates. Our results are consistent with previous studies showing a strong association between CSA-AKI and short-term morbidity and mortality, even in patients with slight changes in renal function^[8,11,12,14,17,18]^.

Despite the large number of studies on CSA-AKI, few articles focused exclusively on patients with preserved baseline renal function. The center of attention on the development of CSA-AKI can hamper assessing the real impact of AKI and may underestimate the risk of patients with preserved renal function, considered naturally at a lower risk when compared to patients with renal dysfunction, but obviously this does not mean there is no risk.

Ramos & Dias^[8]^ evaluated 142 patients with preoperative eGFR ≥ 60 mL/min (calculated by the Cockcroft-Gault equation) and found a 43.66% incidence of CSA-AKI (based on Acute Kidney Injury Network classification)^[19]^ and 83.3% (25 over 30 patients) of non-survivors presenting with CSA-AKI. Cho et al.^[11]^ analyzed the association between postoperative AKI (seven days after surgery), acute kidney disease (AKD) (three months after surgery), and CKD development (12 months after surgery) in patients who underwent HVS. A total of 1,386 patients were enrolled and divided into a preserved baseline renal function group (eGFR ≥ 60 mL/min/1.73 m^2^, n = 1190 [85.9%]) and a pre-existing renal dysfunction group (eGFR < 60 mL/min/1.73 m^2^, n = 196 [14.1%]). AKI occurred in 23.9% of patients with preserved baseline renal function and even with early recovery of renal function within three days, and AKI increased the risk of AKD [OR, 3.21; 95% CI, 1.98 to 5.20] and CKD (OR, 2.86; 95% CI, 1.68 to 4.86). Compared with patients without AKI, patients with AKI had significantly greater incidences of major adverse kidney and cardiac events three and 12 months after surgery and significantly higher mortality rates at postoperative months three (29.7% *vs.* 3.6%) and 12 (32.4% *vs.* 4.5%) (*P*<0.001 for all)^[11]^. Charytan et al.^[20]^ also evaluated patients with preserved baseline renal function and found an AKI incidence of 46.6%, 24.3%, and 12.8% in patients with severe, moderate, and without significant CKD, respectively, and severe AKI requiring RRT occurred in < 1% of patients with normal baseline function. Operative death was significantly more frequent in patients with severe CKD (7.1%) and moderate CKD (4.8%) than in patients with no/mild CKD (2.2%)^[20]^.

In a cohort of 931 patients, Chonchol et al.^[18]^ evidenced 817 (87.8%) patients with no/mild CKD (eGFR ≥ 60 mL/min/1.73 m^2^). During the entire follow-up period, 32.5% patients in the CKD group and 19% in the group without CKD met the primary outcome, a composite of death, nonfatal acute coronary syndrome, secondary coronary revascularization, nonfatal stroke or transient ischemic attack, and peripheral vascular surgery (*P*<0.001)^[18]^.

In an article previously published by our group evaluating 2,804 patients who underwent cardiac surgery, a subgroup analysis based on baseline eGFR showed that AKI staging remained an independent predictor of death, with robust results in patients with previously preserved renal function [hazard ratio; 3.08, 17.51, and 48.86 for stages 1, 2, and 3, respectively; *P*<0.001 for all^[14]^.

Our study provides primary evidence that AKI in patients with preserved baseline renal function is a strong predictor of morbidity and early mortality after cardiac surgery, having a dose-response relationship between AKI stages and hospital mortality up to 30 days. This finding suggests that the increase on mortality in patients who developed CSA-AKI occurs not only in patients with previous renal dysfunction, but also in those with preserved baseline renal function. This fact corroborates the importance of early detection of patients at risk of developing CSA-AKI and its prevention and highlights, as shown in several studies, that even slight changes in renal function have a significant impact on outcomes and mortality in patients undergoing cardiac surgical procedures^[12,21-25]^ - even in cases with a preserved baseline renal function^[8,11,14,18,20]^.

### Limitations

This study has several limitations. Firstly, this is a single-center study with a retrospective analysis of prospectively collected data. Thus, the study design did not permit the characterization of potential causes of postoperative AKI, such as hemodynamic, electrolyte, and acid-base disturbances and the use of nephrotoxic or vasoactive drugs. Secondly, the selection of the preserved renal function population might not be representative of the general cardiac surgical population. The interpretation and comparison of the results obtained in the present study with those of studies not discriminating the baseline renal function and based on different AKI classifications might be impaired. Thirdly, cardiogenic shock/vasoplegic syndrome data were not available and were not included in the regression analysis. Finally, despite the use of adjusted regression models, the possibility of confounding factors cannot be completely excluded.

## CONCLUSION

Age, obesity, left ventricular dysfunction, previous cardiac surgery, and CPB duration were identified as predictors of CSA-AKI in patients with preserved baseline renal function. The development of AKI was an independent predictor of hospital mortality up to 30 days after cardiac surgery, even in patients with slight changes in renal function. CSA-AKI patients had worse clinical outcomes, and there was a dose-response relationship between AKI stages and hospital mortality.
